# Ground Beetle (Coleoptera: Carabidae) Responses to Cattle Grazing, Grassland Restoration, and Habitat across a Precipitation Gradient

**DOI:** 10.3390/insects13080696

**Published:** 2022-08-03

**Authors:** Evan S. Waite, Gregory R. Houseman, William E. Jensen, Molly M. Reichenborn, Mary L. Jameson

**Affiliations:** 1 School of Life Sciences, Arizona State University, Tempe, AZ 85281, USA; 2 Department of Biological Sciences, Wichita State University, Wichita, KS 67260, USA; greg.houseman@wichita.edu (G.R.H.); mreichenborn21@gmail.com (M.M.R.); maryliz.jameson@gmail.com (M.L.J.); 3 Department of Biological Sciences, Emporia State University, Emporia, KS 66801, USA; wjensen1@emporia.edu

**Keywords:** grassland management, diversity, grazing, ground beetle

## Abstract

**Simple Summary:**

Beneficial insect numbers have sharply declined in recent years, and these declines negatively impact (1) food crops due to reduced pollination services, and (2) wildlife and birds due to reduced food sources. In part, agricultural intensification and habitat fragmentation have led to these declines. In the United States, one conservation effort is the Conservation Reserve Program (CRP), which provides financial assistance for replanting agricultural land to restored habitat to improve environmental health and quality. Common CRP grassland restorations are CP2 (“native grass” seed mix) and CP25 (“rare and declining habitat” seed mix). We examined the response of ground beetles, a group that is important for wildlife, to CRP restoration and management across three grassland habitats, and 108 sites. We examined two restoration types (CP2, CP25), and the grazing or absence of grazing by cattle. Our findings indicate that ground beetle communities are not negatively impacted by moderate levels of cattle grazing. Additionally, we found that cattle grazing might have positive effects on ground beetle abundance, biomass, and diversity in tallgrass habitats. The positive impact of cattle grazing may provide an additional incentive for CRP restorations that would enhance beneficial insect populations.

**Abstract:**

Grasslands in North America have declined by over 70% since industrialization of settlements due to the conversion of natural habitats to cropland and urban centers. In the United States, the federally supported Conservation Reserve Program (CRP) was created to improve water quality, reduce soil erosion, and increase native habitats for wildlife. Within these restored grasslands, ground beetles (Coleoptera: Carabidae) are a keystone invertebrate group that fill several crucial niches and may serve as bioindicators of successful land management strategies. To understand the impact of land management on ground beetles, we examined carabid beetle community responses to a grazing treatment and two plant restoration treatments with low and relatively high initial plant diversity over two field seasons. We used pitfall traps at 108 CRP sites across a 63.5 cm precipitation gradient, encompassing three grassland types. Overall, grazing and restoration treatment did not have detectable effects on carabid abundance, biomass, or diversity. Carabid communities, however, responded differently to grazing within grassland types—all three community measures increased in response to grazing in tallgrass sites only. Our short-term study suggests that moderate levels of cattle grazing do not negatively affect carabid communities and might have positive effects on abundance, biomass, and diversity in tallgrass regions.

## 1. Introduction

Numerous reports demonstrate that insect populations are in severe decline due to anthropogenic stressors [1,2,3,4,5], illuminating deep concerns in conservation biology. Habitat loss, fragmentation, and deterioration are recognized as contributing factors for this decline [3]. Habitat restoration can positively influence insect community reassembly [6,7,8], and a focus specifically on insect communities can promote essential ecosystem services such as pollination, pest control, carrion and waste removal, and food resources for other organisms [9,10,11,12]. One key invertebrate group that provides ecosystem services [10], is heavily impacted by human-imposed stressors [13], and responds positively to habitat restoration [14,15] is the ground beetles (Coleoptera: Carabidae). Ground beetles fill several crucial niches within ecosystems as predators, seed dispersers, and prey for other organisms [10]. The wide diet breadth of this beetle group is considered beneficial for reducing pest insects and weedy plant populations, thus allowing native plant communities to thrive [16,17,18,19]. In addition, their role as a food source for other organisms cannot be overlooked. They play a large role in the diets of most vertebrate groups, including game birds, such as pheasant, grouse, and quail, as well as non-game wildlife, such as small mammals, herptiles, and bats [20,21,22,23,24,25].

Ground beetles are microhabitat specialists, inhabiting alpine meadows, tree canopies, mossy logs, leaf litter patches in deciduous forests, sandy riparian areas, and sparsely vegetated grasslands [10]. The habitats required to support ground beetle communities can be altered by land management regimes that lead to changes in carabid community composition, making them useful indicators of environmental change [13,26,27,28]. For example, the removal of tree canopies leads to a decline in the abundance of woodland species, while grassland and woodland edge species increase in abundance [29]. Additionally, intensive disturbance results in communities with smaller, macropterous species [30], while increased time between disturbances allows for colonization by larger-bodied, flightless species [31]. Due to their strict microhabitat associations and responses to change in the environment, carabid beetle species are useful indicators of environmental disturbance.

In North America, some of the most severely disturbed ecosystems are the Great Plains grasslands, which have declined over 70% since industrialized settlement [32]. Over time, development has led to the displacement of native species and ecosystems due to conversion of natural habitats to cropland and urban centers, as well as the encroachment of woody plants [32]. One program in the United States that restores these declining habitats is the Conservation Reserve Program (CRP). Created in 1985, this program financially compensates landowners to replant their agricultural fields with prescribed seed mixes toward the goal of preserving water quality, reducing erosion, and increasing wildlife habitat [33]. Since the program’s inception, over 22 million acres (90,000 km^2^) of land have enrolled in one of 42 conservation practices (CP or restoration types) that have been created to fill various roles in restoring distinct habitats, each with its own unique seed mix and allowable management practices (e.g., disking, haying, interseeding with forbs).

Differences in initial CRP seed mix composition could result in differences in plant diversity and structural variability across CRP sites. Heterogeneity in both physical plant structure and community composition are identified as key components that support more diverse beetle communities [27,34,35]. However, restored CRP lands lack heterogeneity compared to native prairies [36,37,38]. Among these CRP restoration types, differences in seed mix and management practices could create discrete habitats that favor certain invertebrate community structures, including those of carabids. Additionally, higher diversity in plant communities often leads to higher abundance and richness in insect communities [39,40]. Plant diversity on CRP land differs between conservation practice type [41,42], and may affect the insect community composition (including carabid beetles) even in areas with similar abiotic environmental factors.

Another factor that strongly influences plant heterogeneity is herbivores. Historically, the Great Plains were grazed by large herbivores, primarily bison (*Bison bison* L.) [32]. Although large herds of bison are not common today, domestic cattle (*Bos taurus* L.) are a commercially ubiquitous large grazer that could potentially fulfill a similar role in the management of grassland systems. When grazed at similar densities, plant communities between bison and cattle grazed fields are over 80% similar, despite these two ungulates differing in their physical impacts and foraging behavior [43]. Large herbivores can create heterogeneity by preferentially consuming dominant grasses and allowing non-dominant forbs to thrive, in addition to altering plant height through trampling [44,45]. Waste products left behind by these large herbivores increase plant production by adding nutrients back into the system, creating microhabitat islands, and providing food sources for insects such as fly larvae that dwell in the dung [10,46]. Though grazing on CRP has been disincentivized or entirely restricted [47,48], this natural disturbance is crucial to maintaining ecosystem functions such as nutrient cycling and habitat creation in grasslands [49]. By directly impacting insect habitat conditions with dung, and indirectly through changes in the plant community, the presence of grazers on CRP grasslands is likely to benefit the co-occurring carabid communities.

In this study, we examined 108 CRP sites representing four different treatment combinations across a 63.5 cm (45.7–109.2 cm) annual precipitation gradient, including three different grassland types (short-, mixed-, and tallgrass) that roughly correspond to our three study regions. During two growing seasons, we recorded carabid beetle abundance, diversity, and biomass to assess carabid community response to cattle grazing and restoration type, as well as precipitation. The goals of this study are to assess the response of carabid communities (abundance, biomass, diversity) to: (1) moderate grazing by cattle, and (2) differences in restoration type (CP) that can influence plant diversity (low diversity seed mix (CP2) vs. higher diversity seed mix (CP25)). Understanding these invertebrate communities and how land management may affect community composition across a broad precipitation gradient will inform better stewardship of these communities and ultimately the ecosystem services they provide.

## 2. Materials and Methods

### 2.1. Site Selection

As part of a broader three-year study (2017–2019) on various organisms including birds [50,51], arthropods [50], and plants [52], a randomized list of CRP landowners in Kansas was obtained from the United States Department of Agriculture (USDA), which administers the CRP. Eligibility of a site was determined by minimum area requirements for birds (≥35 acres/14.2 ha [53,54]), distance from the next closest site (≥1 km), and the year it was enrolled in CRP (2012 or earlier, and with a contract not expiring before September 2019). We examined the effects of two restoration types, CP2 and CP25, on ground beetle communities in 2017 and 2018. CP2 is a lower diversity initial seed mix that only requires 2 native grasses to be planted, with the goal of establishing permanent native grasslands. This conservation practice includes incentives for adding forbs in the seeding mix, but forbs are not required [47]. CP25 is a higher diversity initial seed mix that requires 5 native grasses and 4 to 10 native forbs or legumes to be included in the seeding mix [55] and focuses on restoring rare and declining habitats for wildlife [41,42].

**Figure 1:**
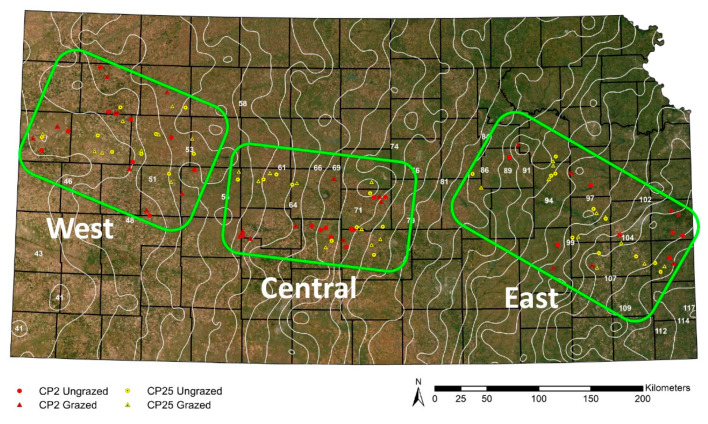
Map of Kansas with satellite image overlay [59] and the location of 108 CRP sites in this study. Background color depicts the precipitation gradient (drier in the west, wetter in the east) and changes in vegetation (short grassland in the west, tall grassland in the east). Red symbols represent CP2 restored sites (lower seed diversity restoration); yellow symbols represent CP25 restored sites (higher seed diversity restoration). Triangles are grazed sites; circles are ungrazed sites. The three study regions are represented by the green boxes (West, Central, East). White lines represent the 30-year average precipitation isoclines [56]. Image by Jackie Baum.

We established three study regions across the longitudinal 30-year average (1981–2010) annual precipitation gradient across Kansas [56]: West (45.7–55.9 cm precipitation), Central (55.9–76.2 cm precipitation), and East (86.4–109.2 cm precipitation). Study regions divided the precipitation gradient into areas of short-, mixed-, and tallgrass grassland that correspond to the west, central, and east regions, respectively (Figure 1). Because the structure of plant communities is heavily influenced by precipitation [49] and affects insect populations and distributional ranges [57,58], this regional approach allowed us to make generalizations about the effect of precipitation and, in turn, the grassland types and beetle communities that correspond to varying levels of precipitation.

In order to understand changes in carabid communities based on our treatments in different grassland types across the precipitation gradient, we examined the representative grassland type in each region. The regions followed the USDA seeding zones prescribed for Kansas [60]. Additionally, we followed USDA grassland designations (i.e., short-, mixed-, or tallgrass grassland) for each county where a study site was located; our western region was primarily short grass, the central region was mixed or sand grass, and the eastern region was mainly tall grass [47,48] (Table S1).

Across all 108 study sites, we balanced the replication of restoration type (18 CP2 and 18 CP25) and grazing (18 grazed and 18 ungrazed) in each region, though there were minor discrepancies in this distribution due to changes in landowner management, as well as lack of available landowners for a given CP willing to participate in the study (Tables S2 and S3). For grazed sites, landowners provided and managed their own cattle at stocking rates determined by the local Natural Resource Conservation Service (NRCS), following NRCS 528 Prescribed Grazing guidelines [61]. These rates were tailored to each individual field with a goal of removing 50% of the available standing plant biomass within a given grazing season. Stocking rates were determined based on cattle type, forage availability and grazing duration, and therefore were not equivalent across sites, but all targeted the same 50% biomass removal goal. Landowners were instructed to allow the cattle to graze 120–180 days between April and October 2017 and 2018. The type of cattle (cow/calf, steers, etc.) was chosen by the landowner, and any type was permitted if it complied with the NRCS grazing recommendation. Because our goal was to evaluate the carabid beetle response to restoration treatment (CP2/CP25) and grazing treatment (grazed/ungrazed), we did not include native, unrestored sites.

Within each of the 108 CRP sites (Figure 1), a 200 × 300 m experimental plot was established as close to the center of the field as possible with the longest axis of the site determining the orientation of the plot (Figure S1). If the property included more than one CRP tract, the plot was placed within the largest field. Fieldwork was conducted with permission of private landowners and as permitted by the USDA.

### 2.2. Insect Sampling

Each experimental plot contained 9 points distributed across three transects, with three points spaced 100 m along each transect and 75 m between each transect (Figure S1). At each site, points 2, 4, 6 and 8 each contained one non-baited pitfall trap (Figures S2 and S3), an effective method to sample ground-dwelling insects [62]. This yielded 432 traps per year (864 total samples). Capture rate of pitfall traps is proportional to activity density of ground-dwelling arthropods and provides relative estimates of abundance. Pitfall traps were made of two 650 mL (22 fl. oz.) semi-transparent plastic cups (diameter: 9 cm, depth: 15 cm), including an outer cup (with holes drilled on the bottom and sides) and an inner cup (with holes on the sides). The cups were nested within one another (Figure S2), with the drilled holes (65 mm from the top of the cup) aligned to allow rainwater to drain into the soil rather than overflow the trap. The inner cup was filled with 180 mL (6 fl. oz.) of a 50:50 propylene glycol and water solution. The trap was placed in the soil so that the top of the trap was flush with the surface of the ground. A metal grate (“pitfall guard”) with diamond-shaped openings (67 × 27 mm), large enough to allow all insects to passively fall into the trap, was placed over the top of the trap and was secured using four landscape staples positioned on each corner (Figure S3). This pitfall guard was designed to prevent the cattle on grazed plots from stepping into traps. Pitfall guards were used on all experimental plots regardless of the grazing treatment.

Pitfall traps were deployed early in the field season (23 May 2017 to 23 June 2017, and 28 May 2018 to 8 June 2018) and then collected after 3–7 weeks (27 June 2017 to 25 July 2017, and 26 June 2018 to 10 July 2018). To reduce variation in the number of days a trap was deployed and to prevent specimen degradation, we narrowed trap deployment from 24–47 days in 2017 (34 average trap days) to 28–35 days (31 average trap days) in 2018. Pitfall traps were retrieved from the field and then processed in the lab.

### 2.3. Laboratory Methods

All organisms collected in the pitfall traps were examined using a dissecting microscope (Leica M80 0.8×–1.0× Achromatic lens, Buffalo Grove, IL, USA, http://www.leica-microsystems.com, accessed on 15 June 2019) and sorted into the following target taxa: Carabidae, Histeridae, Silphidae, Scarabaeoidea, and other Coleoptera. Orthoptera were also sorted (2018 only). Non-target specimens such as arachnids, other insect orders, terrestrial isopods, and decapods were sorted into “by-catch”.

Identifications were made to the genus-level using Ball and Bousquet [63], and species-level using the following keys: *Amara* [64], *Amblycheila* [65], *Anisodactylus* [64], *Brachinus* [66], *Calathus* [67], *Calosoma* [68], *Chlaenius* [69], *Cicindela* [70], *Cicindelidia* [70], *Cyclotrachelus* (as *Evarthus*) [71], *Dicaelus* [72], *Dromochorus* [70], *Euryderus* [73], *Galerita* [74], *Geopinus* [64], *Harpalus* [75], *Harpalus (Megapangus)* [76], *Helluomorphoides* [77], *Micrixys* [63], *Panagaeus* [64], *Pasimachus* [78], *Poecilus* (as *Pterostichus*) [64], *Pterostichus* [64], *Scaphinotus* [79], *Scarites* [80], *Selenophorus* [64], and *Tetracha* [70]. Identifications were facilitated with comparative collections (University of Kansas, Carnegie Museum of Natural History, California Academy of Science, and University of California Berkeley) and with the assistance of Bob Davidson, Dave Kavanaugh, and Kip Will (Carnegie Museum of Natural History (CMNH), California Academy of Science (CASC), University of California Berkeley (EMEC), respectively). Higher classification (subfamily, tribe, genus) follows the classification of Adephaga (Coleoptera) by Bousquet [81].

Carabid specimens occasionally became disarticulated due to water logging. If large pieces of a beetle (i.e., thorax + abdomen, head + thorax) were present and identifiable as Carabidae, but were not able to be identified to species, then these were placed into a category of “unidentifiable”. This category was included in the activity density (hereafter, referred to as “abundance”) and biomass evaluations (because these measures are independent of species identity), but were not included in diversity measures.

Following identification, total carabid beetle biomass from each site was dried for 168 h in a Yamato DKN 810 (Tokyo, Japan, https://www.yamato-scientific.com/, accessed on 15 June 2019), weighed immediately to prevent absorption of ambient humidity using an Ohaus AR1530 balance (Parsippany, NJ, USA, https://us.ohaus.com/en-us/, accessed on 15 June 2019), and recorded to the nearest 0.001 g.

Voucher specimens are deposited at the following public institutions: Carnegie Museum of Natural History (Pittsburg, PA, USA; CMNH), Wichita State University Invertebrate Collection (Wichita, KS, USA; WICHI), California Academy of Science (San Francisco, CA, USA; CASC), and the Snow Entomological Museum Collection at the University of Kansas (Lawrence, KS, USA; SEMC). This research did not include any endangered or protected Carabidae species.

### 2.4. Data Analyses

Carabid species richness (number of species), total abundance (number of individuals of all species), and total biomass (dried weight) were recorded (see Supplemental Data (original data)). Data from all four traps at each site were pooled before analysis. Abundance, diversity, and biomass were similar between years; thus, to examine the overall effects of restoration type (CP2 or CP25) and grazing treatment (grazed or ungrazed) on the ground beetle community, we pooled site data from 2017 and 2018. Site East 32 was an extreme outlier with an incredibly high abundance of carabids (548 individuals: 9.3% of the total number collected, 3.3 times as many beetles as the site with the second highest abundance). There was no clear reason why this site had such a high abundance of carabids. We conducted statistical analyses with and without this site (Table S4). Our goal was to represent the average treatment effects across 108 sites as a way of informing management decisions. One highly atypical site does not accurately reflect that goal, so the results presented excluded this site. Additionally, we examined carabid responses to treatments with and without standardizing the data to account for sampling effort. The length of time each trap was deployed varied between sites and years (see Section 2.2 above); however, the pattern of significance was the same with and without standardization (Table S5 and Figure S4). To provide a clear interpretation, results are discussed based on unadjusted values.

Shannon–Weiner species diversity (H) was calculated for each site and then averaged for each treatment combination within a region (West, Central, East) using the “vegan” package [82] in RStudio [83]. Because Shannon–Weiner diversity is a logarithmic measure and is not easily comparable between studies, we calculated the effective species number (ESN) for our treatments (*e^H^*). This linearized metric is directly comparable between multiple studies [84].

Carabid data were not normally distributed; neither standard transformations (log, square root, 4th root) nor alternative distributions (Poisson, Negative Binomial, Gamma) adequately met statistical assumptions. A generalized linear mixed model (GLMM) approach using Gamma distribution for trap-day adjusted data was explored but returned non-convergent models. Consequently, non-parametric statistical analyses were conducted on beetle abundance, biomass, and diversity. Kruskal–Wallis one-way analysis (package “stats” 3.5.1) [83] was used to determine differences in overall abundance, diversity, and biomass of beetles on our treatment combinations separately per study region. Statistical tests compared levels of a treatment (CP2/CP25, grazed/ungrazed) within each region. Analyses were conducted using R (Version 3.4.3) [83].

## 3. Results

### 3.1. Carabid Beetles

**Figure 2:**
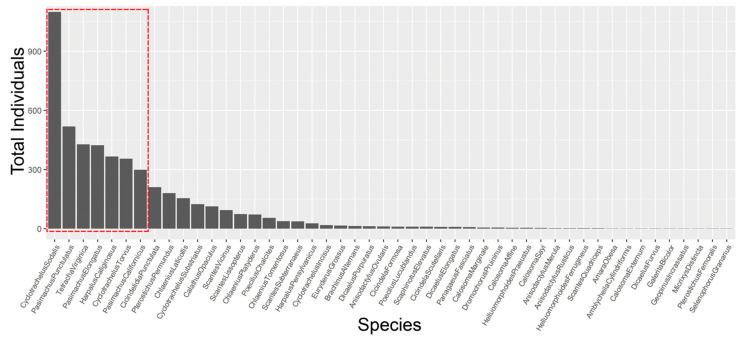
Rank abundance of all 48 restored grassland carabid beetle species from highest to lowest abundance. Seven species (red box) represent 68% of all carabids collected across 108 sites in 2017 and 2018.

A total of 5078 carabid beetles were collected at 108 study sites over two field seasons. The 4821 individuals collected represented 48 species (Table S6; 257 were pieces and unidentifiable to species) across all sites (Figure S5). There were no adventive or non-native species; the invasive and rapidly expanding species, *Pterostichus melanarius* Illiger, was not found. The three most abundant species in each region (seven species total) accounted for 68% of the total number of carabids collected (Table 1 and Figure 2). These species were *Cyclotrachelus sodalis* LeConte (1099), *Pasimachus punctulatus* Haldeman (518), *Tetracha virginica* L. (427), *Pasimachus elongatus* LeConte (424), *Harpalus caliginosus* (Fabricius) (366), *Cyclotrachelus torvus* (LeConte) (355), and *Pasimachus californicus* (Chaudoir) (299) (Table 1, Figure 2). Nine species were represented by single individuals: *Amara obesa* (Say), *Amblycheila cylindriformis* (Say), *Calosoma externum* (Say), *Dicaelus furvus* Dejean, *Galerita bicolor* (Drury), *Geopinus incrassatus* (Dejean), *Micrixys distincta* (Haldeman), *Pterostichus femoralis* (Kirby), and *Selenophorus granarius* (Dejean).

### 3.2. Carabid Abundance

**Figure 3:**
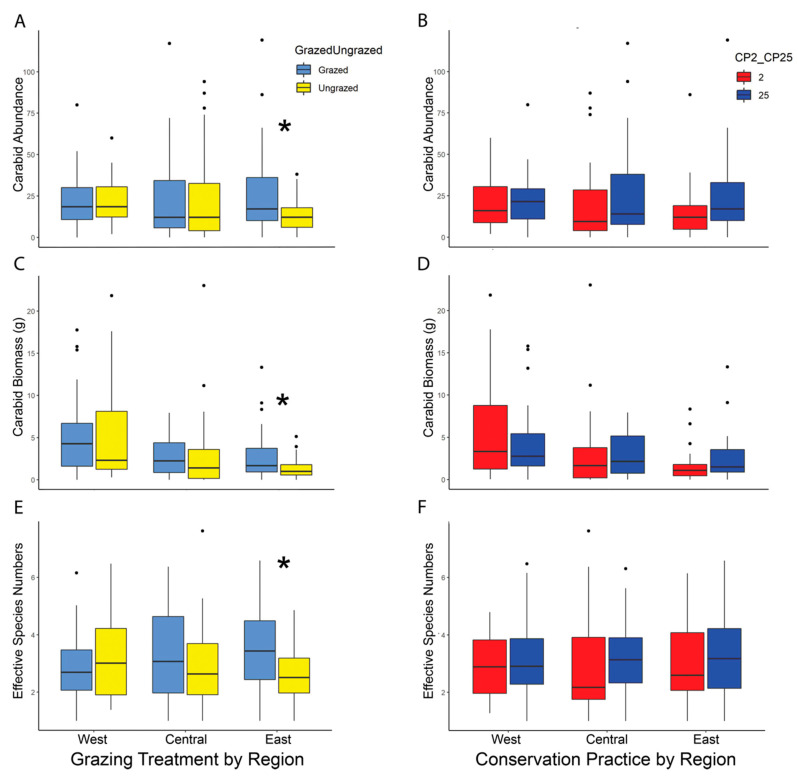
(**A**)–(**F**): Boxplots showing the median carabid abundance, biomass, and diversity by study region (West, Central, East) and treatment (grazed vs. ungrazed, and CP2 vs. CP25). There was no effect of restoration type (CP2 vs. CP25) on these three carabid measures (**B**,**D**,**F**). There was a significant positive effect of grazing on carabid abundance ((**A**), *p* = 0.033), biomass ((**C**), *p* = 0.033), and diversity ((**E**), *p* = 0.010) in the East study region. Boxes represent the middle 50th percentile of data while each whisker represents an additional 25th percentile. The dots represent data points more than 1.5 times outside of the interquartile range. Asterisks used to denote significant *p*-values from Kruskal–Wallis tests (*p* ≤ 0.05). Figures for data adjusted for trap days can be found in the supplemental documents (Figure S4).

Kruskal–Wallis tests showed no significant difference in overall carabid abundance between restoration types (CP25 high diversity initial seed mix or CP2 low diversity initial seed mix) or grazed vs. ungrazed treatments (Table 2). However, there was significantly higher beetle abundance on grazed sites compared to ungrazed sites in the East region (grazed = 927, ungrazed = 467, median 39 vs. 25, respectively) (χ^2^ = 4.53; df = 1; *p* = 0.033) (Figure 3A). There was no significant effect of CP on carabid abundance in any region (Figure 3B).

### 3.3. Carabid Biomass

Similar to the results for carabid beetle abundance, Kruskal–Wallis tests showed there was no overall effect of CP or grazing on beetle biomass (Table 2). However, average carabid beetle biomass was significantly higher on grazed sites than ungrazed sites in the East region (grazed = 98.12 g, ungrazed = 46.40 g, median 3.93 g vs. 2.37 g, respectively) (χ^2^ = 4.49; df = 1; *p* = 0.033) with no differences in the Central or West region (Figure 3C). There was no significant difference in biomass between CP2 or CP25 in any region (Figure 3D).

### 3.4. Carabid Diversity

Ground beetle diversity was similar across the restoration type (CP) and grazing treatments (Table 2). However, there was significantly higher carabid diversity on grazed sites in the East region than on ungrazed sites (ESN = 3.56 vs. 2.69, respectively; χ^2^ = 6.47; df = 1; *p* = 0.010) (Figure 3E). There was no significant difference in diversity between restoration type in any region (Figure 3F).

## 4. Discussion

### 4.1. Response of Carabid Communities to Grazing and CP Treatments

We examined carabid community response to moderate cattle grazing and restoration type across 108 restored grasslands spanning 650 km, with a precipitation gradient of 63.5 cm (139% change in precipitation from the driest to the wettest site on average). Across all 108 CRP sites, we found no consistent overall effect of grazing or restoration type (CP2 vs. CP25) on carabid beetle abundance, diversity, or biomass. Despite equivalent grazing pressures, we only observed a significant positive effect of grazing on the carabid beetle community measures in the East region. This significant positive effect of grazing was not observed in the West or Central regions. There were no regional effects associated with the restoration type (conservation practice or CP).

Similar to effects on carabids due to other grazing mammals [85,86,87], we predicted that grazing would increase carabid abundance, with a concomitant increase in carabid biomass. In addition, grazing may increase carabid diversity [88], thus we expected positive effects across the entire precipitation gradient. However, studies in Norway and Scotland showed that across varying grazing pressure, carabid abundance increased with grazing pressure, but diversity indices were similar across all sites [87,89]. Our results partially corroborate these findings. We observed there was no overall effect on carabid diversity between our grazing treatments (grazing: *p* = 0.212; Table 2); however, grazing significantly increased carabid abundance and diversity in the eastern tallgrass region. This was not due to a larger species pool and associated increase in abundance of carabid species. This differential response in the East region was also seen in plants associated with Kansas CRP [52] and may be driven (at least in part) by the increased mean annual precipitation. The comparatively high mean annual precipitation in the east may create differences in microhabitat availability and plant community structure, thus improving resources for insects. It is worth noting that despite looking at the 30-year averages, precipitation deviated slightly from this average during the study. In 2017, sites experienced average levels of precipitation, but in 2018, several sites experienced severe drought. These annual differences could potentially explain the lack of effect for some variables.

Grazing and precipitation can have combined effects on plant communities [90], which may mediate the response of the carabid community. The combined effect of precipitation and grazing is important to carabids and other insects because cattle preferentially graze certain plants [91], thus modifying the plants in a habitat. Grazing preferences change with plant availability [43], a factor heavily driven by precipitation [92]. Changes in the plant community can incite shifts in the insect community as well. For example, in grasshopper communities in Mongolia, grazing had a significant positive effect on grasshopper diversity across a 20 cm precipitation gradient in various plant communities [93].

The weak relationship between grazing and the carabid community may be explained by the implemented intensity and duration of grazing in this study. After two years of grazing on almost half of our sites (53 of 108 sites), we found that the prescribed stocking rates did not remove the targeted amount of biomass in any study regions, with a mean reduction of only 24% (Table S7), whereas the targeted rate was a 50% reduction. Some insects, including carabids, show a preference for more intensively grazed habitats [87,94]. It is possible that the reduction in plant biomass was not enough to cause a significant shift in the overall ground beetle community. Long-term studies address lag effects of different management strategies [95,96], and the time needed to detect a lag effect may be longer than the two seasons of 120–180 days of grazing that we implemented for this study.

While we were able to standardize grazing pressure across the precipitation gradient, there were numerous factors out of our control, such as the age of each restoration and the frequency at which the sites were burned. Historically, CP2 is the older of the two practices, thus CP2 sites were typically established earlier (25.26 ± 0.90 [SE] yr) than CP25 sites (13.85 ± 0.92 [SE] yr) [52]. Additionally, burning was used as a mid-contract management more frequently in eastern tallgrass sites (33.6% of sites) than in western short grass (1.2% of sites). Because of this disparity in burn frequency and unreliable records of burns outside of the years included in this study, the effects of fire were not examined. A forthcoming publication will examine the interactions between the carabid community and plant structure, plant diversity, percent bare ground, precipitation, treatments, and species turnover across a broad landscape using multivariate analyses.

### 4.2. Carabid Significance in Grassland Restoration and Management

Carabid beetles are used as a bioindicators of habitat type, management success (haying, grazing, etc.), and disturbance in European ecosystems [87,97,98], but there is a lack of research that examines carabid community responses in North America. In Europe, a long history of carabidology has created an excellent taxonomic foundation and understanding of their diverse ecological roles and life histories [87,99,100,101,102,103]. In contrast, the life histories of many North American species remain largely unknown. In this study, we address the gap in knowledge of North American grassland carabids due to few extensive surveys of this fauna. Sampling at 108 sites gives insight into these ground beetle communities on a scale, unprecedented both in the US and in Europe. For comparison, carabid studies in Europe used from 3 to 30 study sites [27,87], and in the United States, from 12 to 44 [85,104]. Previous studies on grassland carabids in the US have focused on the response of carabids to fire, rather than planting or grazing management [105,106]. Despite the current paucity of studies that utilize carabids as indicators of environmental change in the US, long-term carabid beetle data collected by the NEON project (National Ecological Observatory Network) [107] will likely focus future North American research on carabids as essential indicator taxa.

By addressing this gap within US grassland habitats, we provide a baseline for future North American carabid ecology studies including foundational data for comparison of un-invaded carabid beetle communities in Great Plains grasslands systems. *Pterostichus melanarius*, an invasive carabid species that is spreading rapidly in the US, dominates and restructures invertebrate communities [108,109]. Originally from Europe, this invasive species currently is distributed across the northern US and Canada, and southward into neighboring states such as Iowa and Colorado [81]; it is predicted to expand its range into Kansas [110]. It is associated with disturbed habitats [109], thus its likely expansion into restored CRP grasslands might lead to shifts from native carabid communities.

Our results indicate that moderate levels of grazing positively influence carabid abundance, diversity, and biomass in North American tallgrass habitats, and does not negatively impact carabids in short- and mixed-grassland habitats. Similar to the response of the plant community in this research, restoration type (CP2 or CP25) did not have any significant effects on the carabid community [52]. These results assist in informing CRP management strategies, suggesting that moderate grazing can build more diverse and abundant carabid beetle communities. While no management favors all species, grazers are a historical component of grassland ecosystems, and their use could positively impact many ground dwelling insects. One of the primary goals of CRP is to increase the habitat for wildlife that often rely on the invertebrates for food. Managing CRP land in ways that benefit carabid beetles will aid game birds such as pheasant, grouse, and quail, as well as non-game wildlife, such as small mammals, herptiles, and bats that use ground beetles as food [20,21,22,23,24,25]. When management benefits the invertebrate community, the organisms that depend on invertebrates for their ecological services within these ecosystems will stand to benefit as well.

## Figures and Tables

**Table 1:** Most abundant ground beetle species in each region and their abundances. *Cyclotrachelus sodalis* (*) was a dominant species found in all regions. Other species are unique to their region.

West	No.	Central	No.	East	No.
*Pasimachus punctulatus*	468	Cyclotrachelus sodalis *	308	Cyclotrachelus sodalis *	450
Cyclotrachelus sodalis *	341	*Cyclotrachelus torvus*	273	*Tetracha virginica*	377
*Pasimachus californicus*	202	*Pasimachus elongatus*	164	*Harpalus caliginosus*	336

**Table 2:** Kruskal–Wallis results for overall (across regions) carabid beetle community measures, treatments, and year. There were no significant differences between treatments (*p ≥* 0.05).

Carabid Beetle Community Measure	Kruskal–Wallis Output	Treatments Grazed/Ungrazed CP2/25	
Abundance	Chi	2.14	0.959
	df	1	1
	*p*	0.142	0.327
Diversity	Chi	1.319	2.964
	df	1	1
	*p*	0.250	0.085
Biomass	Chi	2.307	1.557
	df	1	1
	*p*	0.128	0.212

## Data Availability

The data presented in this study are available online at https://zenodo.org/record/6774608#.YulcpnbMI2x (accessed 2 August 2022).

## Supplementary Materials

The following are available online at https://www.mdpi.com/article/10.3390/insects13080696/s1, Supplementary Materials 1—Table S1: Distribution of grassland type in each study region; Table S2: Distribution of each restoration type in each study region; Table S3: Distribution of the grazing treatment in each study region; Table S4: Kruskal–Wallis results with outlier site E32 included; Table S5: Kruskal–Wallis results for the east region when standardized for trap days; Table S6: Ground beetle species and their abundances; Table S7: 2018 Total plant biomass difference between grazed and ungrazed sites in each region; Figure S1: Experimental plot design in each CRP site; Figures S2 and S3: Pitfall trap design and pitfall trap in situ; Figure S4: Boxplots showing the median carabid abundance, biomass, and diversity adjusted for sampling period; Figure S5: Species accumulation curve per site for all regions. Supplementary Materials 2—Sheet 1: Metadata associated with data collection and definitions of each field. Sheet 2: Carabid data used for analyses.
