# Supplementary material for: Ground Beetle (Coleoptera: Carabidae) Responses to Cattle Grazing, Grassland Restoration, and Habitat across a Precipitation Gradient
Source: Insects. 2022 Aug 3;13(8):696. doi: 10.3390/insects13080696 (PMC9409295; doi:10.3390/insects13080696)
Supplement: Supplementary file 1 [file insects-13-00696-s001.zip › Supplementary Materials 1.pdf]

Supplementary Document: Ground beetle (Coleoptera: Carabidae) responses to cattle grazing, grassland restoration, and habitat across a precipitation gradient

Citation: Waite, E.S.; Houseman, G.R.; Jensen, W.E., Reichenborn, M.R., Jameson, M.L. Ground beetle (Coleoptera: Carabidae) responses to cattle grazing, grassland restoration, and habitat across a precipitation gradient. *Insects* **2022**, *13*, x. https://doi.org/10.3390/xxxxx

Original data: Ground beetle data for this research are publicly available at https://zenodo.org/record/:XXXXXXXXXXXXXXXXX

Table S1: Distribution of grassland type in each study region

Region	# of Short Grassland Sites	# of Mixed Grassland Sites	# of Sand Grassland Sites	# of Tall Grassland Sites
West	32	4	0	0
Central	0	13	17	6
East	0	0	0	36

Table S2: Distribution of each restoration type in each study region

Region	Number of CP2 Sites	Number of CP25 Sites
West	18	18
Central	18	18
East	14	22

Table S3: Distribution of the grazing treatment in each study region

Region	Number of Grazed Sites	Number of Ungrazed Sites
West	18	18
Central	17	19
East	18	18

Table S4: Kruskal-Wallis results with outlier site E32 included. Bold numbers indicate significant p-values.

Carabid Beetle Community Measure	Kruskal-Wallis Output	Treatments	
		CP2/CP25	Grazed/Ungrazed
**Abundance**	Chi	4.59	2.91
	df	1	1
	p	**0.032**	**0.087**
**Biomass**	Chi	4.41	2.68
	df	1	1
	p	**0.035**	0.101
**Diversity**	Chi	1.3477	4.31
	df	1	1
	p	0.245	**0.037**

Table S5: East region Kruskal-Wallis results when standardized for sampling effort (trap exposure days). The standardized values return the same level of statistical significance as the non-standardized analysis. Graphs to accompany these results are below (Figure S4). Bold numbers indicate significant p-values.

Carabid Beetle Community Measure	Kruskal-Wallis Output	Treatments	
		CP2/CP25	Grazed/Ungrazed
**East Abundance**	Chi	3.68	4.15
	df	1	1
	p	0.055	**0.041**
**East Biomass**	Chi	3.08	4.15
	df	1	1
	p	0.079	**0.041**
**East Diversity**	Chi	0.588	6.14
	df	1	1
	p	0.442	**0.013**
**Central Abundance**	Chi	0.832	0.203
	df	1	1
	p	0.361	0.651
**Central Biomass**	Chi	0.863	2.44
	df	1	1
	p	0.352	0.118
**Central Diversity**	Chi	0.916	1.51
	df	1	1
	p	0.338	0.218
**West Abundance**	Chi	0.038	0.015
	df	1	1
	p	0.843	0.901
**West Biomass**	Chi	0.369	0.426
	df	1	1
	p	0.543	0.5136
**West Diversity**	Chi	0.012	0.441
	df	1	1
	p	0.910	0.506

Table S6. Ground beetle species and their abundances. All individuals were collected in non-baited pitfall traps. Feeding guild information obtained from [10] and [64][1]. Asterisk (*) denotes designation based on congeners due to lack of information on the species.

Subfamily	Species	No.	Feeding Guild
Brachininae	*Brachinus alternans* Dejean 1825	13	Predator*
Carabinae	*Calosoma affine* Chaudoir 1843	4	Predator
	Calosoma externum (Say 1823)	1	Predator
	Calosoma marginale Casey 1897	5	Predator
	Calosoma sayi Dejean 1826	3	Predator
	Scaphinotus elevatus (Fabricius 1787)	11	Predator
Cicindelinae	*Amblycheila cylindriformis* (Say 1823)	1	Predator
	Cicindela formosa Say 1817	10	Predator
	Cicindela scutellaris Say 1823	9	Predator
	Cicindelidia punctulata (Olivier 1790)	211	Predator
	Dromochorus pruininus Casey 1897	5	Predator
	Tetracha virginica (Linnaeus 1767)	427	Predator

Subfamily	Species	No.	Feeding Guild
Harpalinae	*Amara obesa* (Say 1823)	1	Omnivore/Granivore*
	Anisodactylus merula (Casey 1918)	2	Omnivore/Granivore
	Anisodactylus rusticus (Say 1823)	2	Omnivore/Granivore
	Anisodactylus ovularis (Casey 1914)	11	Omnivore*
	Calathus opacalus LeConte 1854	113	Omnivore*
	Chlaenius laticollis Say 1823	157	Omnivore*
	Chlaenius platyderus Chaudoir 1856	71	Omnivore
	Chlaenius tomentosus (Say 1823)	38	Predator
	Cyclotrachelus incisus (LeConte 1846)	29	Omnivore*
	Cyclotrachelus sodalis (LeConte 1848)	1099	Omnivore
	Cyclotrachelus substriatus (LeConte 1846)	126	Omnivore
	Cyclotrachelus torvus (LeConte 1863)	355	Omnivore
	Dicaelus elongatus Bonelli 1813	8	Predator
	Dicaelus furvus Dejean 1826	1	Predator*
	Dicaelus purpuratus Bonelli 1813	12	Predator
	Euryderus grossus (Say 1830)	15	Granivore*
	Galerita bicolor (Drury 1773)	1	Predator
	Geopinus incrassatus (Dejean 1829)	1	Omnivore
	Harpalus caliginosus (Fabricius 1775)	366	Omnivore/Granivore
	Harpalus pensylvanicus (DeGeer 1774)	27	Omnivore/Granivore
	Helluomorphoides ferrugineus (LeConte 1853)	2	Predator
	Helluomorphoides praeustus (Dejean 1825)	4	Predator*
	Micrixys distincta (Haldeman 1852)	1	Likely molluscivore[1]
	Panagaeus fasciatus Say 1823	7	Unknown
	Poecilus chalcites (Say 1823)	54	Omnivore
	Poecilus lucublandus (Say 1823)	10	Omnivore
	Pterostichus femoralis (Kirby 1837)	1	Predator
	Pterostichus permundus (Say 1830)	180	Omnivore
	Selenophorus granarius Dejean 1829	1	Omnivore/Granivore*
Scaritinae	*Pasimachus californicus* Chaudoir 1850	299	Predator
	Pasimachus elongatus LeConte 1846	424	Predator
	Pasimachus punctulatus Haldeman 1843	518	Predator
	Scarites lissopterus Chaudoir 1881	73	Predator*
	Scarites quadriceps Chaudoir 1843	2	Predator
	Scarites subterranaeus Fabricius 1775	36	Predator
	Scarites vicinus Chaudoir 1843	94	Predator
Other	Unidentified Carabidae (parts)	257	
	Total Number	5,078	

Table S7: Percent difference in mean plant biomass between grazed and ungrazed sites in each region (2018). The overall biomass reduction for all regions was an average of 24%. All grazed sites saw lower biomass levels than ungrazed sites in all regions (D.F. Watson *et al. In Prep.*).

Region	Percent Reduction in Mean Biomass with Grazing	Standard Error of the Reduction in Mean Biomass Expressed as a Percent
West	33%	8.67%
Central	15%	5.78%
East	24%	7.18%

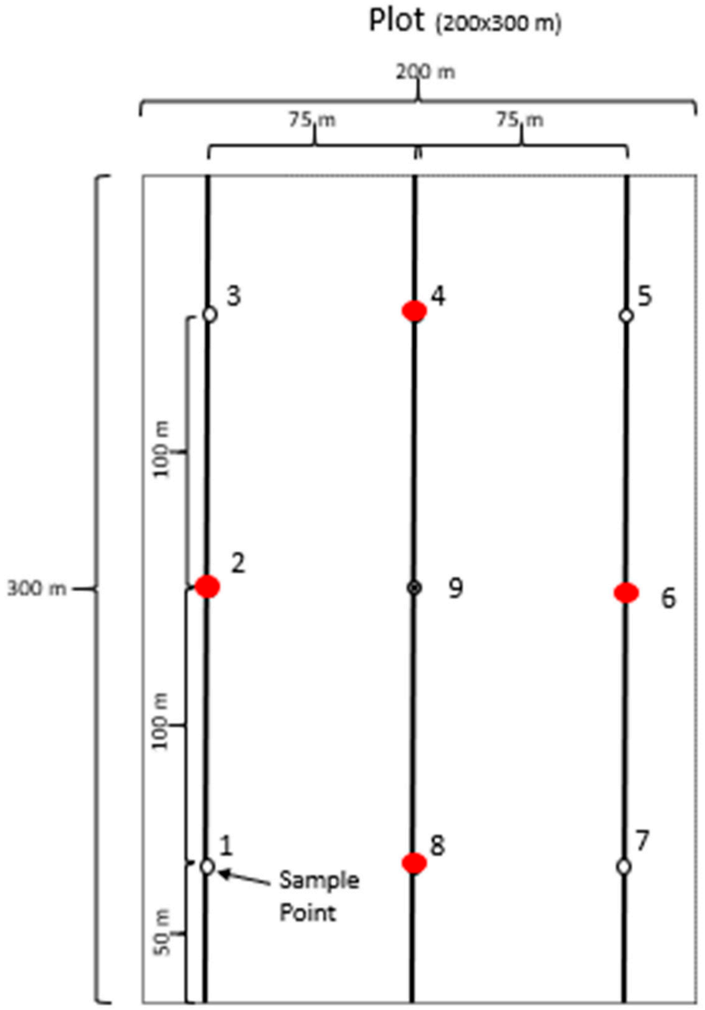

Figure S1. Experimental plot design in each CRP site. Numbered points (1–9) are locations for plant data collection. Points marked in red indicate (2, 4, 6, 8) the placement of pitfall traps for carabid beetle sampling. Image by Gregory Houseman.

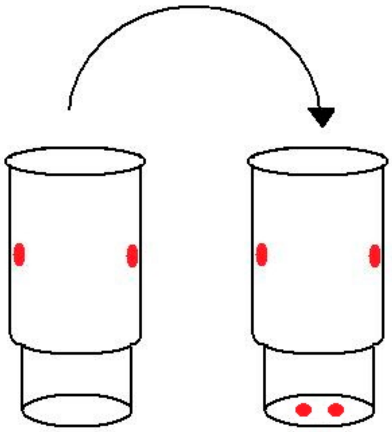

Figure S2. Pitfall trap design. Drilled holes are indicated in red (to allow drainage of rain from the trap). Cup on left (inner cup) is inserted into cup on right (outer cup), and holes are aligned. Placement of holes allowed rainwater to drain into the soil rather than overflowing the trap. Image by Rachel Stone.

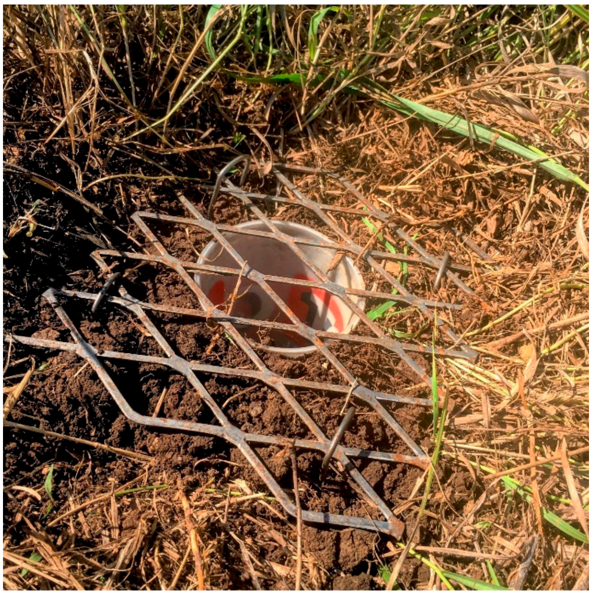

Figure S3. Pitfall trap *in-situ*. Pitfall trap was placed in the ground at plot points 2, 4, 6, and 8 with the rim flush with the soil. On all sites, a pitfall guard was placed on top of the trap and secured with a landscape staple at each corner. The guard prevented injury to cattle on grazed sites. Photo by Evan Waite.

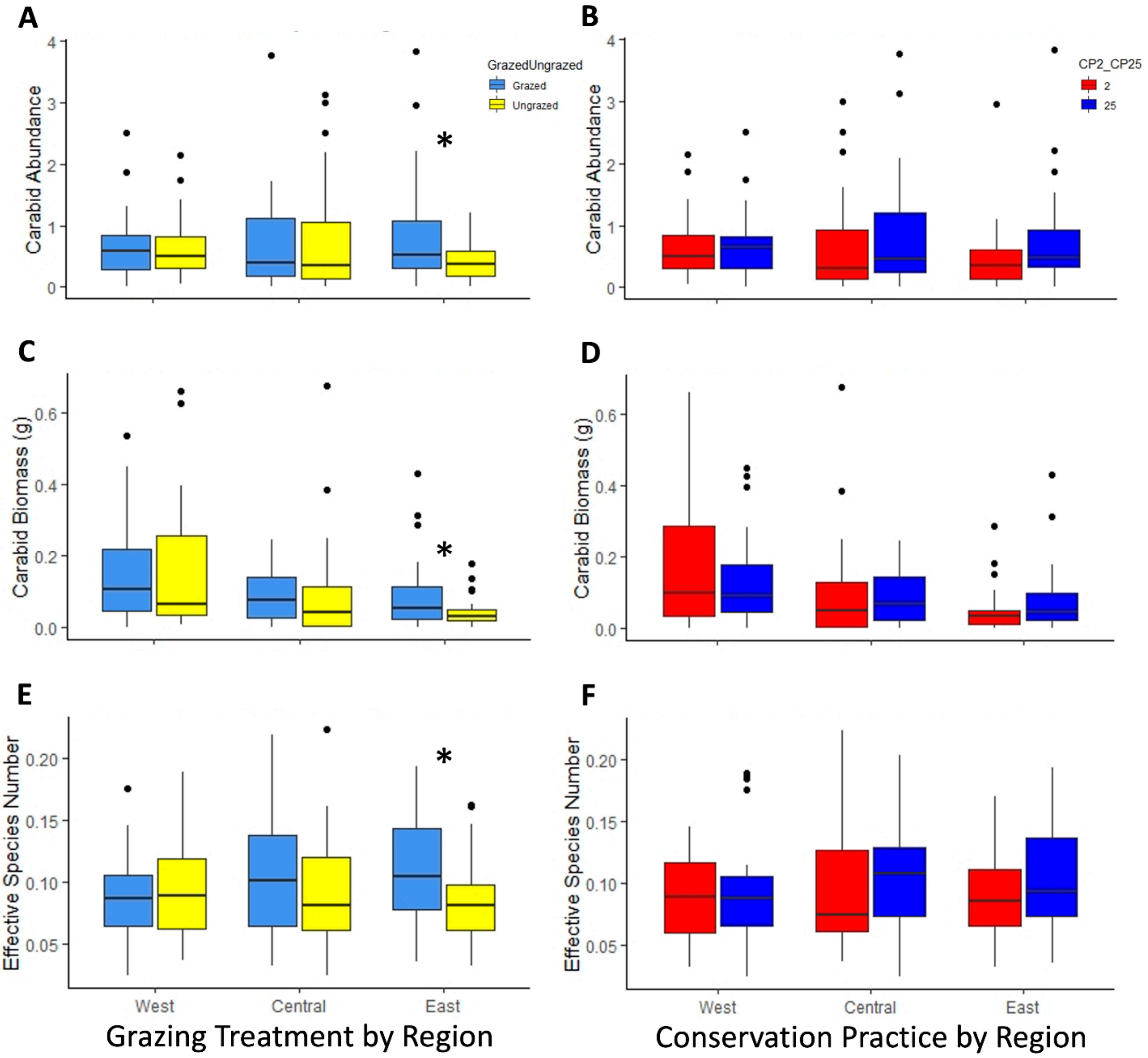

Figure S4: Boxplots showing the median carabid abundance, biomass, and diversity collected per day by study region (West, Central, East) and treatment (grazed vs ungrazed and CP2 vs CP25) and standardized by sampling effort (trap exposure days). This was calculated by dividing the abundance, biomass, and diversity by the number of days the pitfall traps were deployed at each site (trap days). Like the unadjusted data, there was no effect of restoration type (CP2 vs CP25) on these three carabid measures in the Central and West regions (B, D, F). There was a significant, positive effect of grazing on carabid abundance (A, $p = 0.041$), biomass (C, $p = 0.041$) and diversity (E, $p = 0.013$) in the East study region. Boxes represent the middle 50th percentile of data while each whisker represents an additional 25th percentile. The dots represent data points more than 1.5 times outside of the interquartile range. Asterisks denote significant p-values from Kruskal-Wallis tests ($p \leq 0.05$).

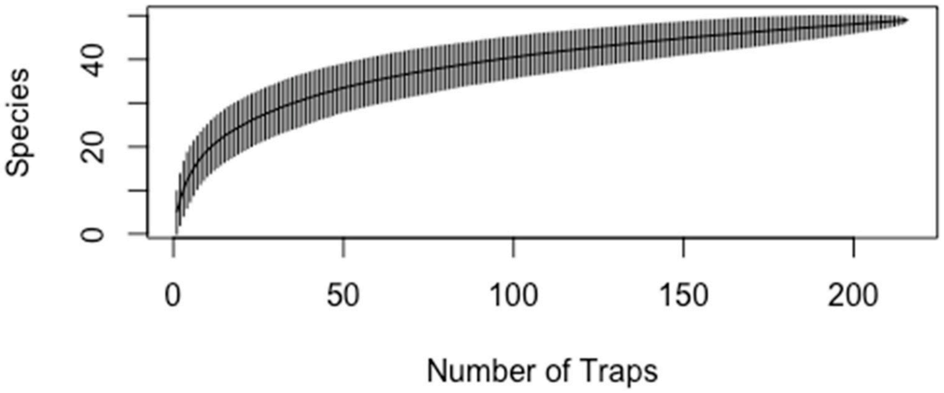

Figure S5. Species richness accumulation curve per trap (site) for carabid species collected across all regions. The curve represents the successively pooled species; the vertical lines are the standard deviation of 1000 permutations. The curve was computed in vegan.
